# Spontaneous Combustion

**Published:** 1851-08

**Authors:** 


### WESTERN JOURNAL

OF

### MEDICINE AND SURGERY.

AUGUST, 1851.

ART. I.—*Spontaneous Combustion.* Translated from the Archives Générales de Medicine. By P. K.

A celebrated trial which recently took place in the capital of the grand duchy of Hesse Darmstadt, and summarily reported in the March number of the Archives Générales, de Medicine is of such scientific and legal importance that we feel justified in giving it a more or less extensive publicity in our country.

On the 13th June, 1847, at about eleven o'clock P. M., the partially consumed body of the Countess of Goerlitz was found in her chamber in the midst of burnt and burning furniture. The apartment of the countess consisted of three pieces, viz: a principal chamber, with antechamber and cabinet; the first mentioned of which was lighted by windows upon the court yard, and the second upon the street. The apartment could only have been entered by breaking open the doors upon the interior, of which the keys were not found. At the time when the murder must have been committed and the fire kindled, the countess was alone in the house with a servant named Strauss, (who was not for a long time accused of the crime,) and she could not possibly have received assistance. The count of Goerlitz was dining at the ducal palace and did not return until eleven o'clock, the hour at which the calamity, accident, or murder was discovered. The countess was accustomed when remaining alone, to lock the doors of her rooms and take out the keys. At three in the afternoon she was seen for the last time in the enjoyment of her usual health. She was a woman not more than forty-six years of age, at the most, very active in her habits, not fat, and by no means addicted to the abuse of alcoholic drinks, and consequently fulfilling none of the conditions observed amongst those who are usually the victims of what is called spontaneous combustion. Ordinarily she was blessed with good health, and though delicate, was not subject to nervous affections, and seemed by no means to be exposed to apoplexy or syncope by which a fall might have been induced and an accidental burning caused. Towards eight P. M., the occupants of the house on the opposite side of the way, observed in the chamber of the countess a bright light similar to one caused by a fire in the fire place, which lasted for a quarter of an hour, and they also remarked a thick, black smoke which seemingly issued from the chimney corresponding with the cabinet. At eleven o'clock, when the count returned from the palace, there existed no external signs of combustion. At the moment, however, of opening the doors of the apartment, it was seen filled with a thick smoke of an empyreumatical and suffocating odor, and the secretary, perceived to be burning, broke immediately into a flame setting fire to the window curtains. Near this secretary, some few feet from the door-way, the body of the countess horribly disfigured was found stretched upon the floor. The body was lying in the direction of the window with the legs towards the cabinet, the two extremities were together and slightly flexed. The garments were thrown as high as the knee. The body was lying upon the side, with the arms slightly stiffened, it was immediately removed to another part of the house. The leaf of the secretary—the part generally used in writing—the upper and lower drawers were almost entirely consumed, and particularly the lower ones ; the remainder of this piece of furniture was only partially destroyed. The flooring in front of and beneath the secretary was burnt through the extent of some twenty inches, and through to the joists.

But the flooring of the spot occupied by the dead body exhibited no appearances tending to indicate that a burning body had been placed upon it. The mirror placed above the sofa was broken in several places, and was covered with a coating which was shown by chemical analysis to be organic matter, the product of the carbonization of vegetable and animal matters. The bell rope was found upon the floor, burnt at its upper extremity, just below where it was fastened, and at a considerable distance from the secretary. In the cabinet there was a divan, in the middle of which was burnt a hole about a foot and a half in size, and destroying the covering, the hair and moss with which it was filled, and the girthing supporting it. An oil painting hung above the divan was so thickly covered by a coating similar to that covering the mirror, that its colors had entirely disappeared ; near this divan was found the slipper of the countess, which was missing when the body was carried to an adjoining room. The candlesticks containing candles were in the places usually occupied by them, and nothing seemed to indicate that the countess had used them or any other light.

The body of the countess, examined in the chamber whither it had been transported, presented the following appearances :—

The head and neck, the anterior part of the thorax, and the superior extremities from the fingers to the shoulder were horribly burned, but the clothing which covered the lower part of the body was not touched by the fire. The head was reduced to the size of one's double fist, and everywhere equally burned. The remains were of a deep brown color with a greasy brilliancy like a coating of varnish. The mouth was wide open, and the tongue completely carbonized. Some of the experts thought there had been propulsion of the tongue. The neck, like the head was burned in its entire circumference, presenting a similar appearance, but having lost less of its volume. The burn extended as far upon the back as could be seen without turning the body. Upon the anterior surface of the body, the burn extended over the thorax as far as the pit of the stomach ; thence it ran in the form of an arc over the two sides of the thorax, in such manner that the unburnt portions of the garments, and of the body were almost on the same line. At the inferior part of the chest, where the burn was confined to the integuments, the skin for the extent of about an inch, puffed and was slightly carbonized towards its edge.—Above this point and in front, the clothing and the tissues of the body were all so uniformly burned with the exception of the sternum, the clavicles, the ribs and intercostals, that the integumentary covering of the breast and the muscles of the chest seem to have been removed by a knife. This surface was also of a deep brown color, but less lustrous than the head. The arms seemed to be uniformly carbonized from the tips of the fingers to the shoulders. But the tissues were recognisable, they were deeper colored than the other burnt parts, being perfectly black. The head of the left humerus protruded directly upwards about three inches. One of the persons who assisted in moving the body, declared that, having taken it under the arm, it seemed to him at that instant it was the arm bone left the shoulder, and that there remained in his hand only one soft and fatty mass. There was no redness, no true vesicles—some of the witnesses testified to having seen them, but their presence was not observed by the physicians. No wound, no proof of violence, preceding the burning was discovered.

At first the difficulty of explaining so extraordinary a case, joined to the total absence of all traces of violence upon the body of the countess, gave rise generally to the belief of the spontaneousness of the combustion. Dr. Sieboldt was especially of this belief, and it was not until the commencement of November--five months after the accident--that a judicial inquest was commenced against a servant of the late countess named Strauss, some of the jewels of the countess having been discovered in the possession of his relatives. However, the spontaneous-combustion party still rejoiced in its partizans. In a memoir, dated April 12, 1848, ably composed, but in which gratuitous suppositions and subtle reasonings supplied the place of solid inductions, Dr. Sieboldt took the position that it was impossible to explain otherwise the circumstances connected with, and the amount of the combustion. He explained the divers marks of fire in the apartment, by supposing that it had been communicated to the furniture from the body of the countess. For instance lying wrapt in sleep upon the divan of the cabinet, spontaneous combustion might suddenly have commenced in the head, and thus that portion of the divan upon which the head rested has been destroyed; afterwards rushing into her bedchamber and falling near the secretary, she might have thus communicated the fire to this and other articles of furniture, etc. On the other hand, Dr. Graff, in a report made on the 14th of July following, in the name of the medical college of the Grand Duchy, held the opposite opinion. The author who believes in the possibility of spontaneous combustion of the human body, arrived, by comparing the different circumstances of this case with the reports of cases of spontaneous combustion, at the conclusion that the countess of Goerlitz's case was not one of the latter. According to him the most essential requisites for spontaneous combustion were wanting, and he refuted most satisfactorily the suppositions by which Dr. Sieboldt explained the details of the catastrophy. One of the arguments upon which Dr. Sieboldt especially relied was drawn from the difference of the incineration of a body under ordinary circumstances, and upon the insufficiency of the combustibles consumed in the apartment of the countess for producing the effects observed. Dr. Graff thought the reverse with reference to the carbonization of the soft parts of the body, and of the flat and thin bones, and he cited as proof the following, as he thinks, analogous case. The part of the table upon which the head of the body rested was very near the door of a stove containing a lighted fire, and under this table were scattered shavings. The odor of smoke was perceived, and when the cause of it was discovered the shavings and upper parts of the table were found consumed by fire, as was also the head of the body, in place of which remained a shapeless, disgusting carbonized mass. Such an appreciation of the facts in the case led Dr. Graff to believe that the death of the countess of Goerlitz was most decidedly caused by assassination. He desired the exhumation of the dead body for the purpose of examining, if possible, the condition of the bones of the head, for he believed that not strangulation alone, but that fracture of the skull had also been resorted to.

The body was exhumed on the 11th August, 1848, 14 months after the accident, but without any very important disclosures. It established the carbonization of most of the bones of the head, and the complete destruction of more than two thirds of them, principally of the left side, the carbonization of the head of the left humerus, the destruction of the contents of the thorax, except some traces of the heart and diaphragm, and the preservation of the belly which had but very slightly putrefied, etc. On the right temporal bone, almost perpendicular to its superior edge, there was a slight fissure half an inch long, but this might be easily accounted for either by the action of fire, or by direct violence. It was established that the head was the part which had most suffered by the combustion, it being almost entirely carbonized, whilst on the neck, breast and arms, the soft parts only were destroyed by the combustion. This accorded strictly with the superficial examination of the corpse when the traces of the effects of fire gradually diminished from the head towards the pit of the stomach.

The College of Medicine, together with the physicians who had previously testified, concluded that the case was one, *not* of spontaneous combustion, but of incension, for the purpose of concealing a murder accomplished by means of choking and fracture of the skull. Dr. Sieboldt still sustained his first opinion.

In the midst of these conflicting opinions, the presiding judge, shortly before the trial, desirous of procuring all possible light upon the subject, directed Profs. T. Bischoff and J. de Liebig, to be summoned, in conjunction with the College of Medicine, and the experts previously named, for the purpose of resolving the knotty points arising in the case.

It is impossible and unnecessary to reproduce here the report of the new committee. Suffice it to say, that they unanimously rejected the supposition of spontaneous combustion, on the ground of its absolute impossibility, and that Dr. Sieboldt himself, ultimately gave in to this opinion. The cause of this decision was developed in an oral examination of Messrs. Bischoff and Liebig, and to this we will refer further along. As to the other questions bearing upon the cause of the death of the countess, and upon the effects of the fire, we append the following brief extract from the report:

1st. The countess of Goerlitz did not fall a victim to the effects of an external fire, and was not exposed, either of her own accord, or through the instrumentality of others, to such a frightful accident. Death from such a cause does not seem possible, unless the individual falls into the midst of an intense fire which completely envelops him, as a burning house for instance, or without he be so disabled that he cannot save himself, or finally the fire seizes upon the clothing, especially that of females which is particularly favorable to the propagation of the flames. In the case in question neither of these circumstances, but rather the reverses occurred. Besides, it is impossible that a sane person should voluntarily seek death by the slowest and most painful of all means. And it is equally difficult for a single individual to compel another to perish by means of a slow fire. There is, moreover, nothing indicative of the action of fire upon the living body—the vesicles, to the existence of which one witness testifies, being most imperfectly proved.

2d. It is certain that the body of the countess was exposed to the action of fire after her death. But information derived immediately after the calamity is so very incomplete, so unsatisfactory, that it is impossible to decide with certainty what was the cause of the death, before she was subjected to incension. Subtile poisons, such as strychnine, prussic acid, morphia, etc., might have been employed and fire resorted to, for the purpose of concealing the traces of the poison. But this presumption is opposed by all physical and moral circumstances, and so much time has elapsed since the death of the countess, as to render it impossible to discover any traces of such poison, as alone could have been used. It is scarcely more probable that she could have fallen under the effects of a sudden attack of illness, or some unfortunate accident, and then have been accidentally consumed by fire. We are forced to conclude, not only by exclusive but also by direct reasoning, that the countess of Goerlitz was murdered, and that fire was used for the purpose of concealing the crime. The protrusion of the tongue is not sufficient evidence of strangulation, when we take into consideration the degree of burning of the hard and soft parts of the face, and the different action which fire has upon parts which are free, and upon those which are more or less deeply placed. As to the fissure, observed after the exhumation upon the right temporal, and which the medical college thought might be the point of departure of an extensive fracture of the base of the skull, it might also very readily be the result of the action of fire, as was proved by the existence of similar fissures upon the head, burned during the experiments made at Giessen. Besides numerous other fissures precisely similar to this first one, caused simply by handling, have made their appearance upon other parts of the skull. There is then nothing forced in the deduction that one of these fissures was observed the day of the exhumation, and that the others have been caused from time to time. Nevertheless, it is more probable that the countess was killed by a blow, than that she was strangled, for it is not likely that a man of medium strength, could choke to death without great resistance, a woman, who, like the countess, was robust and accustomed to bodily exercise, unless he had surprised her during sleep, which in this case is an inadmissible supposition.

3d. The committe were divided upon the question whether the incension of the body of the countess was caused solely by the burning of the secretary, or if the fire originated otherwise. The majority thought it both possible and probable that the upper part lay within about 2 feet of the burning piece of furniture. The time during which the fire of the secretary might have acted upon the body,

(three hours and a half,) was sufficient to produce those effects observed. The position of the corpse was favorable to its combustion. The form of the burn on the body, and the clothing, went to show that it had happened with the body in the place and position which it occupied when found. Moreover, there were no marks serving to indicate even that the corpse of the countess had been burnt elsewhere than in her apartment. It is also impossible to admit that the combustion had been caused in the apartment by means of alcohol, oil, charcoal or wood, for it would have been impossible for one man to make the necessary preparations for such a proceeding, such as kindling the fire and keeping it kindled, moving the body, supporting the head and chest, and arranging the arms particularly as they must have been to be burnt, intentionally, as they were, etc. It also seemed impossible for a man to remain in the chamber where the body and clothing were burning, and where it was impossible to open the doors or windows without betraying the fact. That alcohol could not have been employed was proved first by the fact of the quantity which it would have required, a quantity too great to have been carried without exciting observation, (3 litres, about 6 pints and a half, being necessary for the head, and much more for the chest and arms); but then again it would have required a large reservoir for the purpose of exposing the body to the flames. During the combustion of even these three litres a living man could not have remained shut up in the apartment in an atmosphere of which the oxygen would be reduced from 21 in the 100 to 14 in the 100---lower than in expired air. The time too at the disposition of the murderer, (from 7 to 8½ o'clock,) was not sufficient to permit those adjuvants to be found, arranged, used, and all traces of them to be removed. As to the burn on the divan in the cabinet, it might have happened by accident, or been intended to remove blood spots, or to extend the fire.

The minority of the committee, without denying that the combustion of the secretary was sufficient to account for the amount of burning of the body, did not think such was the case from the following circumstances: the singularity of the different circumstances connected with the affair, the absence of all traces of fire in the place where the burnt body was found, and the fire on the divan which had been observed for more than a quarter of an hour from the neighboring house.

We stated that professors Bischoff and Liebig had explained to the court the reasons convincing the majority of the committee of the impossibility of spontaneous human combustion ; as that is the most interesting and important part of the whole we will dwell somewhat upon it.

The first recorded case of spontaneous combustion, said Mr. Bischoff, dates back about one hundred and fifty years (that of Millet a woman of Reims, in 1725.) By human *spontaneous combustion*, we understand that a man has been more or less burned, without our being able by external circumstances to explain the burning. Then it is that we say, this man has burned not by aid of external combustibles, but within himself (spontaneously) ; an expression, which, implying an entire theory, seems to be incorrect. How much better it would be to say ; we do not know how this person died, but, in saying that he died from spontaneous combustion, we only substitute an explanation quite as absurd. Thus the doctrine of spontaneous combustion, which has crept into the science is the result of ignorance, and not of scientific research or experimentation. Upon what authorities does this pretended fact rest? The 45 or 48 cases which have been published, relate to individuals burnt either where there was too little, or an entire absence of, combustible material, to account for the combustion. With the exception of two cases of complete and four of partial consumption by fire, the victims of which survived, no one possessing scientific knowledge, no witness of any sort, has been present during the accident. The first case of complete spontaneous combustion, was witnessed and described by a chambermaid and an unknown person, and the second by a stranger. In the four cases of partial combustion, it is very evident that the first one was caused by burning sulphur, which the burnt person spread upon his clothing in the endeavour to extinguish the flames with his hands; the second was the case of a young girl of Hambourg, upon whose hands there appeared a flame which was followed by vesicles; but this fact though reported by a distinguished physician was not witnessed by himself, or by any other person. Most probably then these vesicles were the result of some disease, or perhaps of a burn intentionally made, for the purpose of attracting attention, of gaining admission into the hospital. The third case, which is very generally known, relates to a priest, named Bertoli, and was reported in 1786, by an Italian surgeon. Any unprejudiced person who reads the account of this case, must be convinced that that individual was burnt by fire communicated to his clothing from a lamp. The fourth is still less worthy of confidence, it relates to a blacksmith named Reynaleau: a narration unconfirmed by the slightest evidence, full of contradictions, and winding up with the characteristic trait, that holy water only was able to extinguish the flames. Not one of these cases has ever been reported immediately after its occurrence by any one of recognized authority. Physicians have arrived in several instances after the catastrophe; and some of the cases have been subjected to a judicial inquest, and have the appearance of reality. But for an observer accustomed to criticism, and to the requirements of rigid observation, the reports which we possess are far from presenting the guaranties indispensable to an enlightened investigation. Phenomena such as those of spontaneous combustion require a methodical observation, which the cases mentioned do not possess the slightest trace of. In none of them was there even an autopsy made, still less a serious, scientific investigation, or chemical analysis. In all the cases of spontaneous combustion which have called forth judicial or medical inquests, we constantly see an exhibition of levity, ignorance, prejudice, credulity, and very often of culpability. At the epoch when most of these facts were reported, science itself was not sufficiently far advanced to supply the lights necessary for an analysis of what was observed. As to the numerous cases that have been reported from hear-say, they are based upon the authority of the school-master, the curate, or the village mayor, but have never given rise to an inquest of any kind. A recent and very remarkable instance will demonstrate how, gradually, these accounts become introduced into the annals of science. M. Bischoff here cited the case of pretended spontaneous combustion, published by the *Gazette des Tribunaux*, and reproduced in the *Journal des Débats*, for February 24th, 1850. The case was that of a man who, in a drinking shop, where he had, according to custom, drank deeply, introduced into his mouth in consequence of a bet, a lighted candle; he was suddenly set on fire from within, and his head and the upper part of his chest were carbonized in half an hour in spite of all the assistance rendered. The death and the effects of the fire were reported to have been verified by two physicians. From information acquired by M. Liebig from different savans of Paris, and especially from the prefect of police, it turns out that the whole account was imaginative, and pure fiction invented for the columns of the paper which had inserted it.

The question of spontaneous combustion has been treated of, adds M. Bischoff, by illustrious men, Rudolphi and Treviranus, MM. Kopp and Nasse. These savans have investigated and have expended a great deal of labor and of science for the purpose of explaining this phenomenon. But incredible as it may seem, without first assuring themselves of the truth of reports as made, they have admitted them as they were presented. Their explanation only could be important, and this is wanting. We do not deny these cases because we cannot explain them, but because their existence must be based upon explanations which tend to overthrow the laws, heretofore admitted as true and exact, of physics, physiology, chemistry and pathology. I will confine myself to the mention of the fact, recognized by all, that a body containing twenty five per cent. of water, does not take fire of itself, and does not continue to burn when started. Suppose we collect all the solid parts of the body, the bones, the skin, the tendons, the muscles, and put the water contained in the body into a vase; kindle these solid parts and their entire consumption will not afford sufficient heat to vaporize the water. Alcoholic excess however, we are told, brings about a modification in the human body which renders possible its spontaneous combustion. It is true, all accounts of cases of spontaneous combustion tell us that the subjects were addicted to the abuse of ardent spirits; it is also true that alcohol is inflammable; and why not admit that the body soon becomes impregnated with it, and that, especially when fire is communicated from the exterior, it can burn. Such reflections may be made, but for the naturalist and physician they are valueless. The knowledge which we possess upon the passage of substances from the stomach and intestines into the blood, teaches us that alcohol reaches slowly and in very small quantities the sanguineous system, and as the circulation goes on with great rapidity, the alcohol is carried almost immediately into the lungs, where the blood comes in contact with the air. There the elements of the alcohol become modified by its combination with the oxygen of the air, forming carbonic acid and water which the respiratory process eliminates from the system. Thus under ordinary circumstances we cannot even prove the presence of alcohol in the blood, since it is decomposed and thrown off by the lungs. Here it may be thought that alcohol taken abundantly will penetrate the blood in substance and in large quantities, and spread itself throughout the whole system. Observations and experiments by distinguished men have been made upon this subject, but they are contradictory; some pretend to have found alcohol in the blood, and even in the brain of persons addicted to drink and who have died drunk. Percy discovered traces of it in the brains of dogs into whose veins he had injected a considerable dose. But the observations of Percy are in direct contradiction to those of Dr. De Pommer, of Zurich, who found no traces of the spirit in the blood. MM. Bouchardat and Sandras were unable to discover alcohol in any secretion exeept in the pulmonary exhalation. So far then it remains to be proved that the human body can imbibe alcohol like a sponge; moreover it is, *a priori*, impossible to admit that the body being saturated with alcohol life could continue for a single instant. The coagulation of the albumen, the arrest of the circulation, and the destruction of the nervous system, are the immediate results of the injection of any considerable quantity of alcohol into the blood of an animal. For my own part I am convinced that a dead body does not become combustible from being saturated with alcohol. I have taken parts of a dog into whose arteries I had injected alcohol at 92°, and they would not burn when exposed either to a flame or to the action of carbon; in the latter case only they roasted and carbonized, but ceased even that so soon as withdrawn from the action of the fire. M. Bischoff ended by refuting and ridiculing the stories of flames issuing from the mouths of drunken individuals.

M. Liebig, who six years since, had given his opinion, upon spontaneons combustion, (*Annales de Chimie et de Physique*, 1844, t. 1, page 331,) likewise opposed, for very extensive reasons, the possibility of the facts reported on the subject. It will be borne in mind, said he, that the idea of spontaneous combustion arose at a period when all opinions upon the subject were erroneous. What takes place in combustion was only known some seventy years ago, (Lavoisier). What is necessary for the combustion of a body, has been known only forty years, (Davy.)

Since the occurrence of the case of Millet of Rheims to the present time, some forty-five or forty-eight cases have occured which are alike in—1st. always having taken place during the winter season; 2nd. the persons attacked have all been drunkards drunk at the time; 3rd. they have most generally happened in countries where rooms are heated by open fire-places, and furnaces of charcoal, in England, France and Italy; in Russia and Germany, where stoves are principally used, deaths from spontaneous combustion are very rare; 4th. there has been no eye-witness to the combustion; 5th. no physician, amongst all those who have attempted to explain these cases, has seen one; 6th. there is no information as to the quantity of combustible matter consumed; and 7th. some time has always elapsed from the commencement of the combustion until the body has been found consumed. M. Liebig also argued with great power upon the principal details reported as connected with spontaneous combustion, and upon the explanations given of them. He also demonstrated clearly the error into which the partizans of the spontaneous combustion theory have fallen. The arguments which they employ being deduced, contrary to all logical rules, in the cases in point; death and the destruction of the body, the cause of which is unknown, being assumed as proof of the truth of the assumed cause. These cases are explained by the fact of the possibility of spontaneous combustion, the existence or possibility of which is proved by the same cases. This discussion we are disposed to believe does not admit of refutation, and gives a fatal blow to the doctrine of spontaneous combustion.

The trial, which occupied the greater part of the month of March, 1850, concluded by the condemnation of J. Strauss, as the murderer of the Countess of Goerlitz, to perpetual imprisonment. The journals have recently published that he has made a full acknowledgment of his crime. He admitted that going into the chamber of the countess upon some household duty, and finding no one, he could not upon seeing that the secretary contained money and articles of value, resist the temptation to steal. The countess having discovered him in the act, he seized and strangled her with some difficulty; then having placed the body in an arm chair, near the secretary, he surrounded it with combustibles, to which he set fire for the purpose of concealing his crime. P. K.

